# Genome-Wide Association Study of Local Thai Indica Rice Seedlings Exposed to Excessive Iron

**DOI:** 10.3390/plants10040798

**Published:** 2021-04-19

**Authors:** Reunreudee Kaewcheenchai, Phanchita Vejchasarn, Kousuke Hanada, Kazumasa Shirai, Chatchawan Jantasuriyarat, Piyada Juntawong

**Affiliations:** 1 Department of Genetics, Faculty of Science, Kasetsart University, Bangkok 10900, Thailand; reunreudee.k@ku.th (R.K.); fscicwj@ku.ac.th (C.J.); 2 Rice Department, Chatuchak Bangkok, 10900, Thailand; phanchita.v@rice.mail.go.th; 3 Department of Bioscience and Bioinformatics, Faculty of Computer Science and Systems Engineering, Kyushu Institute of Technology, Fukuoka 820-8502, Japan; kohanada@bio.kyutech.ac.jp (K.H.); shiraikazum@gmail.com (K.S.); 4 Omics Center for Agriculture, Bioresources, Food and Health, Kasetsart University (OmiKU), Bangkok 10900, Thailand

**Keywords:** indica Thai rice, excessive iron, GWAS, RAR1, Hsp90

## Abstract

Excess soluble iron in acidic soil is an unfavorable environment that can reduce rice production. To better understand the tolerance mechanism and identify genetic loci associated with iron toxicity (FT) tolerance in a highly diverse indica Thai rice population, a genome-wide association study (GWAS) was performed using genotyping by sequencing and six phenotypic data (leaf bronzing score (LBS), chlorophyll content, shoot height, root length, shoot biomass, and root dry weight) under both normal and FT conditions. LBS showed a high negative correlation with the ratio of chlorophyll content and shoot biomass, indicating the FT-tolerant accessions can regulate cellular homeostasis when encountering stress. Sixteen significant single nucleotide polymorphisms (SNPs) were identified by association mapping. Validation of candidate SNP using other FT-tolerant accessions revealed that SNP:2_21262165 might be associated with tolerance to FT; therefore, it could be used for SNP marker development. Among the candidate genes controlling FT tolerance, *RAR1* encodes an innate immune responsive protein that links to cellular redox homeostasis via interacting with abiotic stress-responsive Hsp90. Future research may apply the knowledge obtained from this study in the molecular breeding program to develop FT-tolerant rice varieties.

## 1. Introduction

Rice (*Oryza sativa* L.) is one of the most important cereal grains as more than half of the world’s population consumes it as a staple food. In recent years, rice productivity has declined due to global climate changes. This creates a global need for new stress-tolerant and highly productive rice varieties.

Iron (Fe) is an essential micronutrient controlling multiple biological processes, including chlorophyll biosynthesis, chloroplast development, cellular respiration, nitrogen metabolism, and redox enzyme functions in plants [1,2,3,4,5,6]. Nevertheless, Fe toxicity (FT) stress is common in many rice-growing countries worldwide. Fe could become toxic when accumulated in higher quantities. FT could potentially reduce rice production by 12–49% depending on the genotype, intensity of the stress, and soil nutrient status [7]. In the worst-case scenario, severe FT in rice seedlings can result in complete crop failure [8].

Flooding and waterlogging create low oxygen and reductive environments that reduce ferric ion (Fe^3+^) to ferrous ion (Fe^2+^), which can be quickly taken up by the root and translocated to the shoot via transpiration [8]. Eventually, the large amount of Fe^2+^ accumulation can enhance the Fenton reaction to produce reactive oxygen species (ROS), such as hydroxide (OH-) and hydroxyl (OH·) radical [9,10,11,12]. These radicals can damage cellular components, cause membrane damage and cell death via oxidation of lipid, protein, nucleic acids, and other macromolecules [13,14,15,16]. The most common symptom associated with FT is forming necrotic brown spots on leaves, known as leaf bronzing from oxidative stress damages [17].

One of the economically sustainable ways to enhance rice production under FT is the breeding of tolerant varieties. However, breeding efforts for developing FT-tolerant varieties have been limited since FT tolerance in rice is complex with numerous genes involved. Moreover, the molecular mechanisms and genes controlling FT tolerance in rice are not fully understood. Based on previous reports, rice Fe homeostasis may be divided into root and shoot-based approaches [17,18]. The tolerance in roots involves Fe exclusion and retention using aerenchyma and enzymes to oxidize Fe^2+^ to Fe^3+^, which inhibit transportation from root to shoot [8,17,19,20]. The tolerance in shoots involves Fe storage within the ferritin proteins [18,19] or by vacuolar iron transporter [21] and antioxidant system by several antioxidant molecules and enzymes [19,22].

Several studies have attempted to identify candidate genes conferring FT tolerance for rice breeding. Previous reports of quantitative trait loci (QTL) studies for FT tolerance in rice were performed using DNA molecular markers with a bi-parental population [17,23,24,25,26,27,28,29,30,31,32,33,34,35]. Putative QTLs for leaf bronzing had been frequently located on chromosome 1 as a major region for shoot tolerance [17,23,24,31,32,33,34,36,37]. In addition, many QTL studies that evaluate biochemical, morphological, and physiological phenotypes for FT tolerance only showed minor effects [36,38]. The drawback of the QTL mapping is that only allelic diversity, which segregates between the parents of the particular F_2_ cross, can be assayed, and the amount of recombination can limit the mapping resolution [39]. A genome-wide association study (GWAS) has been recently used as an alternative method to provide insights into the genetic architecture of the traits underlying the natural phenotypic variation [35,37,38,40]. There are several GWAS of FT tolerance in rice with different populations, growing stages, and traits. First, Matthus et al. (2015) conducted a GWAS for FT tolerance at the vegetative stage based on leaf bronzing score (LBS) phenotypes of 329 Asian rice accessions; they identified the glutathione-S-transferases gene as a candidate locus for FT tolerance [37]. Second, Meng et al. (2017) identified genetic regions associated with FT tolerance in MAGIC rice populations’ seedlings using growth and dry weight data [38]. The strongest association regions were located on chromosomes 1 and 3, which were close to the LBS-based QTL identified by Wan et al. (2005) [32]. Lastly, Zhang et al. (2017) performed a GWAS for FT tolerance of 222 indica rice at the seedling stage; they found the strongest association with FT tolerance index on chromosome 2 [35].

Thailand is among the top three of the world’s largest rice exporters, with an extensive collection of diverse rice germplasm from irrigated, rainfed lowland, deep water, and upland ecosystems [41,42]. Chakhonkae et al. (2012) analyzed the level of genetic diversity and structure of 43 Thai rice accessions selected from all rice ecologies and 57 rice accessions with desirable agronomic traits obtained from the International Rice Research Institute (IRRI). They found that Thai and IRRI germplasms were significantly different [41]. Thus, Thai rice accessions could be used as valuable genetic resources for trait improvement. As of the present, there is no reported GWAS for FT tolerance conducted on rice accession from Thailand. Therefore, Thai rice accessions’ enormous genetic diversity could benefit GWAS of genetic architecture underlying FT tolerance.

This study aims to identify candidate loci for FT tolerance in seedlings of a highly diverse Thai rice population. Phenotyping data were applied to GWAS using 41,178 indica SNPs to identify candidate SNP linked to FT tolerance. Finally, selected candidate SNPs were further verified for the SNPs’ presence in other Thai rice accessions with FT tolerance phenotype.

## 2. Results

### 2.1. Phenotype under Fe Toxicity Stress

**Figure 1:**
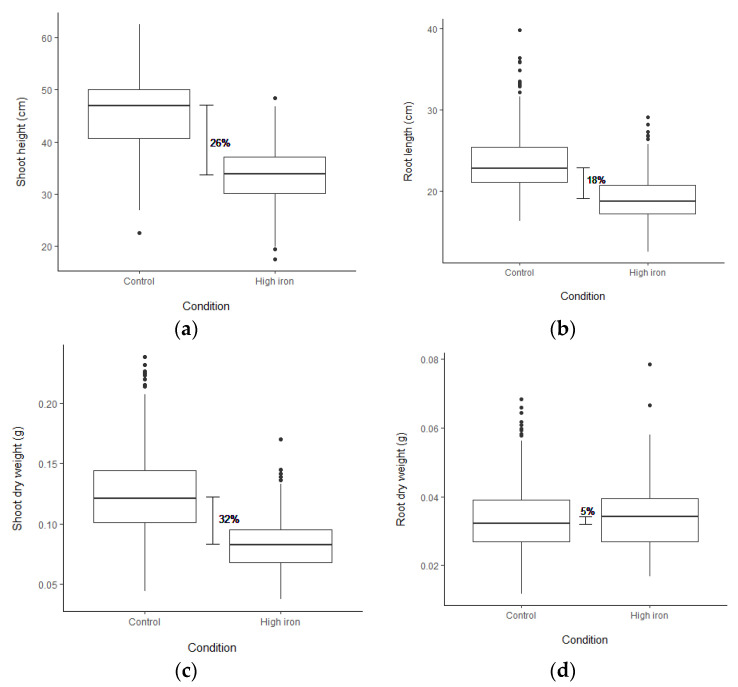
Box plot showing the differences in plant growth and biomass among 270 accessions as (**a**) shoot height, (**b**) root length, (**c**) shoot dry weight, and (**d**) root dry weight. The phenotypes were measured at the seedling stage after applying (1000 ppm Fe^2+^) for five days in both control and high Fe conditions. The horizontal line in the box represents a median of each trait in the condition.

To test for FT tolerance variation, 1000 ppm Fe^2+^ was applied to rice seedlings. The accessions used in this screen comprise 240 Thai rice accessions and 30 representative accessions, including 22 selected RPD1 accessions (Table S1). After three days, stress symptoms, including LBS and chlorophyll contents (SPAD values), were obtained (Figure S1; Table 1; Table S2). Based on LBS, 29 accessions were highly tolerant (LBS = 0–1), while 24 accessions displayed sensitive phenotype (LBS = 5–9) (Figure S2a). The RPD1 accessions, Taichung Native 1 and Kasalath, demonstrated highly tolerant and sensitive phenotypes, similar to the results reported by Matthus et al. [37]. IR64 was sensitive, and Azucena was moderately tolerant to FT (Figure S2a). Nipponbare and Pokkali were more tolerant than Kasalath (Figure S2a). Under FT, the chlorophyll contents of 18 accessions increased more than 20% (Figure S2b). The shoot height (SH), root length (RL), shoot dry weight (SDW) and root dry weight (RDW) were obtained after five days of stress (Table 1; Table S2). Strong growth depression was observed under stress (Figure S2c–e). FT reduced SH, RL, and SDW by 26, 18, and 32%, respectively (Figure 1a–c). In contrast, the average RDW was slightly increased by 5% under stress (Figure 1d). Concomitantly, 146 accessions displayed an increase in RDW under stress (Figure S2f).

To test whether the observed phenotypes resulting from the interaction between genotypes and treatments, the phenotypic data, except LBS, were analyzed by ANOVA. The results revealed that all phenotypic data were affected by genotype, treatment, and the interaction of both (Table 1). Altogether, these results indicated that the phenotypic variation that occurs during stress depends on genotypes. Therefore, the phenotypic data can be used in correlation analysis and association mapping.

**Figure 2:**
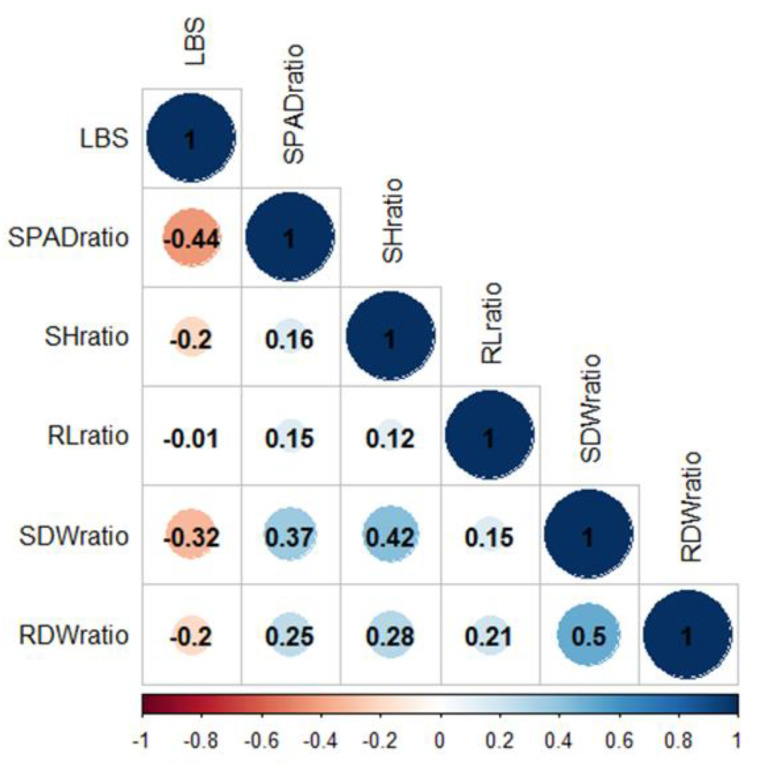
Pearson’s correlation coefficients for phenotypic correlation. Color bar represents correlation value from −1 (red) to 1 (blue). LBS, leaf bronzing score; SPADratio, the ratio of SPAD; SHratio, the ratio of shoot height; RLratio, the ratio of root length; SDWratio, the ratio of shoot dry weight; RDWratio, the ratio of root dry weight.

The relationships between LBS and other ratio values were determined by Pearson’s correlation (r) (Figure 2). LBS demonstrated a strong negative correlation with the SPAD and SDW ratios (r = −0.44 and −0.32, respectively). Linear regression suggested that 19 and 11% of the LBS variation were explained by SPAD and SDW, respectively (Figure S3a,b). The median values for LBS (2.1), SPAD (0.98), and SDW (0.67) ratios were incorporated into the linear regression plot (Figure S3a,b). Among the genotypes with LBS below the median (the more tolerant half of the population), the majority had SPAD and SDW ratios above the median. These data demonstrated that the tolerance accessions could retain chlorophyll and shoot biomass when grown in excess Fe conditions, suggesting that this population’s primary tolerance mechanism could be the shoot-based approach.

### 2.2. Population Analysis and Association Mapping

**Figure 3:**
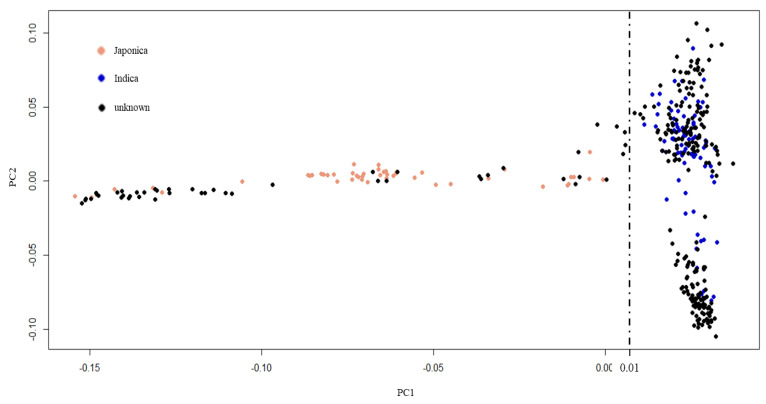
Principal component analysis of population structure. Black dots represent 270 Thai rice accessions. Blue and pink dots represent indica and japonica accessions from 3KRGP, respectively.

Indica rice is the major type of rice grown in tropics and subtropics, including Thailand [43,44,45]. To select an appropriate reference genome for GWAS, our population of 270 Thai rice accessions was compared with rice accessions from the 3000-rice genome project (3KRGP) [46] (Figure 3). First, 1503 core SNPs with less than 0.05 missing data were selected from the Thai rice accessions. These Thai rice core SNPs were used to search with the core SNPs of 3KRGP. Finally, 130 rice accessions (80 indica and 50 japonica) from the 3KRGP that contain the 1301 core SNPs were obtained. The core SNP data of the 270 Thai rice accessions and the 130 3KRGP accessions were combined and analyzed by principal component analysis (PCA). The results demonstrated that the 270 Thai rice accession could be separated into two subpopulations: 222 indica and 48 japonica by PC1 = 0.01 threshold (Figure 3). Therefore, we applied indica reference genome for read mapping and SNP calling.

**Figure 4:**
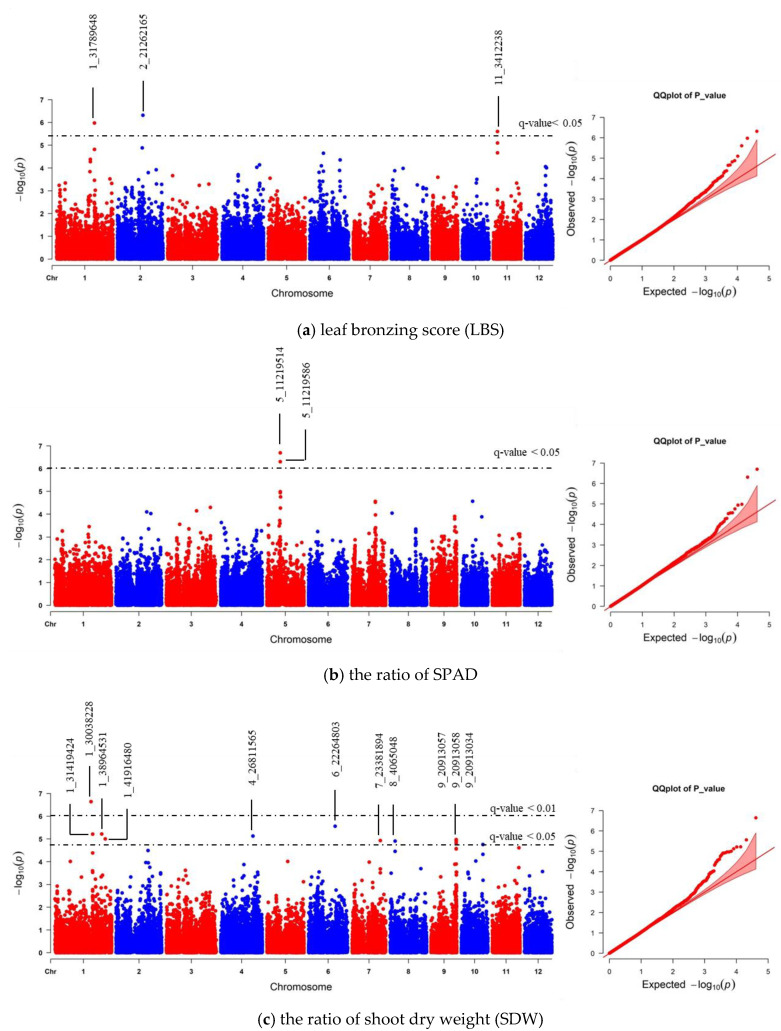
Manhattan and quantile-quantile (Q–Q) plot of genome-wide association study (GWAS) using 41,178 indica SNPs derived from 229 accessions: (**a**) leaf bronzing score (LBS), (**b**) the ratio of chlorophyll content of expanded leaf that was measured by chlorophyll meter SPAD), and (**c**) the ratio of shoot dry weight (SDW). The dashed horizontal line in each Manhattan plot represents the boundary of *q*-value < 0.05 or 0.01 threshold. For each significant SNP, position in the chromosome was displayed.

**Figure 5:**
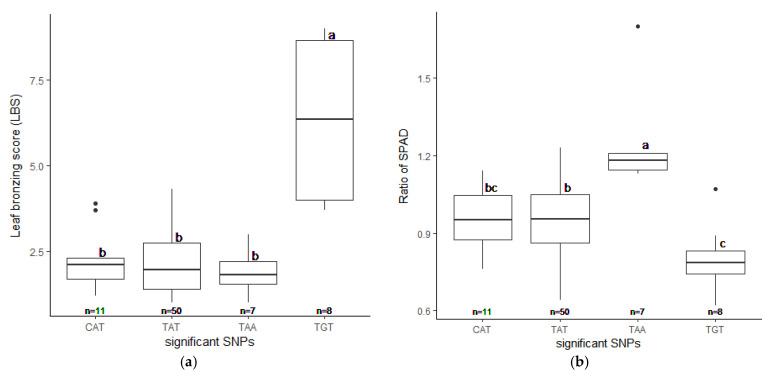
Phenotypes of selected accessions (76 out of 229) with different candidate SNP combinations. The highest significant SNPs, SNP:1_30038228, SNP:2_21262165, and SNP:5_11219514 yield four detectable combination as CAT, TAT, TAA, and TGT. The LBS (**a**) and the SPAD ratio (**b**) were plotted with standard errors. Letters show significant differences between groups at *p* < 0.05 by Tukey’s HSD test.

We realigned the DNA sequencing data using the 93–11 indica genome as a reference genome based on population analysis results. Subsequently, the indica 73,054 SNPs were called, and PCA was performed to obtain the population structure. The results revealed two main groups: group 1 consisted of 229 accessions (indica type), and group 2 consisted of 41 accessions (japonica type) (Figure S4). To minimize the effects from population structure, group 2 data were removed from our panel. The group 1 SNP data (41,178 indica SNPs) were used for association mapping. Our results showed that the average indica SNP density is 108.65 SNPs/Mb (Figure S5). GWAS was conducted by factored spectrally transformed linear mixed models (FaST-LMM). Association mapping results of each trait were shown in Manhattan and quantile-quantile (Q–Q) plots (Figure 4 and Figure S6). SNPs with a *q*-value lower than 0.05 were considered significant. Our results demonstrated that three significant SNPs found on chromosomes 1, 2 and 11 (SNP:1_31789648 (T/A), SNP:2_21262165 (A/G), and SNP:11_3412238 (C/T)) were associated with LBS (Figure 4a; Table 2). There were two SNPs on chromosome 5 (SNP:5_11219514 (T/A) and SNP:5_11219586 (G/A)), which were associated with the SPAD ratio (Figure 4b; Table 2). These two SNPs were haplotypes (TG and AA). One highly significant SNP on chromosome 1 (SNP:1_30038228 (T/C); *q*-value < 0.01) was associated with the SDW ratio (Figure 4c; Table 2). We also found ten other significant SNPs (*q*-value < 0.05) associated with SDW ratio (Figure 4c; Table S3). The Q–Q plot of expected and observed *p*-values from the GWAS is presented in Figure 4. No significant SNP was associated with SH, RL, and RDW ratio (Figure S6). The highest significant SNPs of each trait, including SNP:1_30038228 (T/C), SNP:2_21262165 (A/G) and SNP:5_11219514 (T/A), yielded eight combinations; however, only four forms (CAT, TAT, TAA and TGT) were detected. Our phenotypic data revealed that TAA accessions were FT-tolerant with the average LBS and SPAD ratio of 1.9 and 1.2, respectively (a, b). The most sensitive group was TGT, with the average LBS and SPAD ratio of 6.3 and 0.8, respectively (Figure 5a,b). In contrast, accessions with CAT and TAT combinations were not significantly different from each other based on our LBS and SPAD ratio data (Figure 5), suggesting that SNP:1_30038228 (T/C) could not be used as a marker for FT tolerance. Therefore, only SNP:2_21262165 (A/G) and SNP:5_11219514 (T/A) were considered candidate SNP markers for FT tolerance.

**Figure 6:**
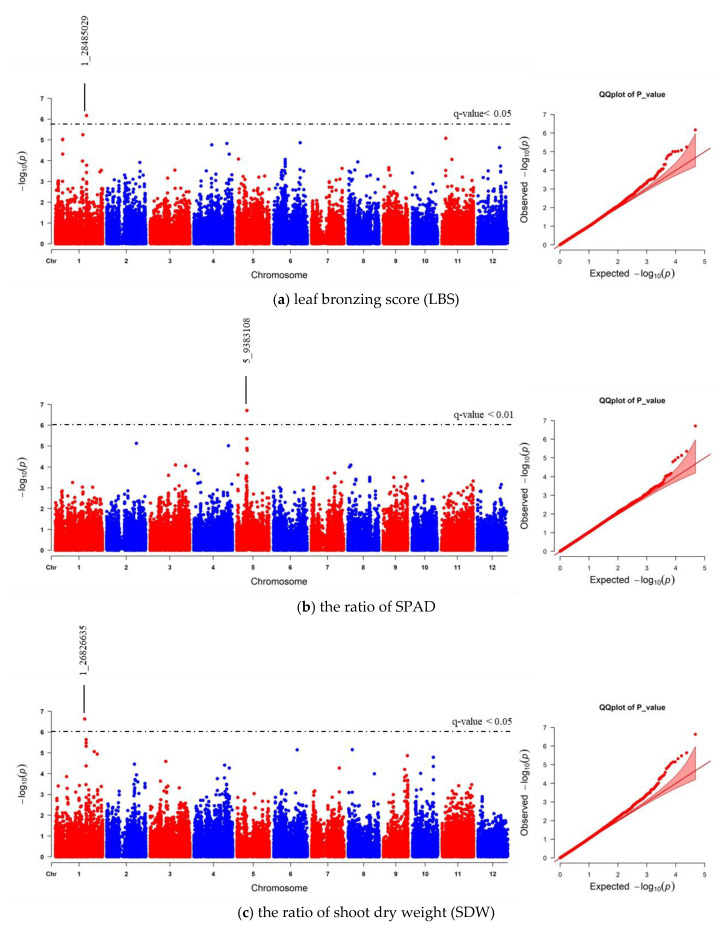
Manhattan and quantile-quantile (Q–Q) plot of GWAS using 47,772 japonica SNPs derived from 229 accessions. (**a**) leaf bronzing score (LBS), (**b**) the ratio of SPAD, and (**c**) the ratio of shoot dry weight (SDW). The dashed horizontal line in each Manhattan plot represents the boundary of *q*-value < 0.05 or 0.01 threshold. For each significant SNP, position in the chromosome was displayed.

We also compared the GWAS results between indica and japonica SNPs data. Our data revealed that the association of LBS and SNP:2_21262165 was lost with using japonica SNPs (Figure 6a; Table 2; Table S3). Instead, SNP:1_28485029 was the only significant SNP related to LBS that could be found using japonica SNPs. This SNP position is located in the same region of previously identified QTL and GWAS [23,37]. Moreover, the number of significant japonica SNPs associated with SPAD and SDW ratios was less than that of the indica SNPs. However, each trait’s highest significant SNPs were still to be found (Figure 6b,c; Table 2; Table S3). We further confirmed that the GWAS results identified, based on indica and japonica SNPs, were correlated using EnsemblPlants comparative genomic tools. Synteny analysis between indica and japonica subspecies showed that all significant SNPs found on the same chromosome were located on the same syntenic regions (Figures S7–S9). All significant SNPs’ data of indica and japonica can be found in Table S3.

### 2.3. Candidate Genes Associated With FT Tolerance in Thai Indica Rice

**Figure 7:**
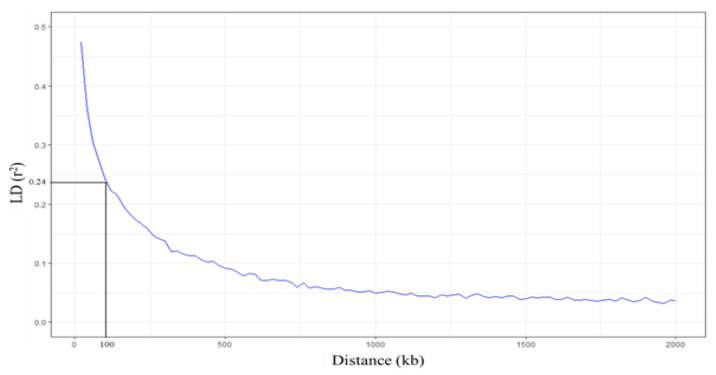
Linkage disequilibrium (LD) decay plots. The *x*-axis represents the distance (kb) between SNPs, and the *y*-axis represents the LD value (*r*^2^). Horizontal and vertical lines represent half LD and LD decay distance, respectively.

Linkage disequilibrium (LD) decay was analyzed within 2000 kb by PopLDdecay [47]. When the LD (*r*^2^) was averaged in every 20 kb, the highest *r*^2^ was 0.47. Our results showed that when the distance at which the average *r*^2^ is halved the maximum value, the LD decay of group 1 is 100 kb (Figure 7). This LD decay range is similar to the previously reported LD decay of indica rice [48,49]. According to the LD decay, genes in the LD block (±100 kb) were searched from the EnsemblPlants database (https://plants.ensembl.org/Oryza_indica (accessed on 18 December 2020)) [50]. We found seven candidate genes located in the LD block of 16 significant SNPs (Table 3; Table S4). *BGIOSGA006309* and *BGIOSGA006308*, found in the LD block of SNP:2_21262165, function in carotenoid biosynthesis and defense response, respectively. SNP:1_31789648, which is located in the intron of *BGIOSGA000995*, encodes kinase domain-containing protein. Other candidates in this region are *BGIOSGA000987* and *BGIOSGA0004247*, which are involved in the glutathione process and chloroplast accumulation/avoidance movement, respectively. Interestingly, *BGIOSGA000987* is *LOC_Os01g49720*, which is a candidate FT-tolerant gene in japonica identified by Matthus et al. (2015) [37]. In the case of SNP:11_3412238, most of the genes found in this LD block are kinase domain-containing proteins (Table S4). *BGIOSGA034416* found in the LD block of SNP:11_3412238 encodes coatomer subunit beta (COPB) protein involved in intracellular protein transport or vesicle-mediated transport. The SNP:5_11219514 and SNP:5_11219586, associated with the SPAD ratio, have one candidate gene, *BGIOSGA019494* (Table S4). This gene encodes thioredoxin domain-containing protein, which operates on cell redox homeostasis. The last candidate gene found in the LD block of SNP:1_30038228 is *BGIOSGA004143.* This gene encodes for aldehyde deformylating oxygenase (ADO), which functions in the lipid biosynthetic process (Table 3; Table S4). These results suggest that detoxification and translocation most likely contributed to FT tolerances in this population.

### 2.4. SNP Validation in Other Thai Rice Accessions

To validate whether the candidate SNP is involved in FT tolerance, we evaluated the candidate SNPs’ presence in other FT-tolerant rice accessions. We searched another set of the Thai rice collection’s genotype by sequencing (GBS) data (200 accessions) and selected 30 accessions with candidate SNP combinations as AA, AT, and GT. Based on the GBS data, 12 and seven accessions were found for the AA and GT groups, respectively. We then randomly picked 11 accessions with the AT genotype to make a complete set of 30 samples. Population structure analysis revealed that the selected 30 accessions were grouped with indica type (group 1), suggesting they could be used as representative accessions (Figure S10). We focused on LBS since it appeared to be the most common symptom of FT stress in rice. Thirty Thai rice accessions were phenotyped in control and high iron hydroponic conditions at the seedling stage.

**Figure 8:**
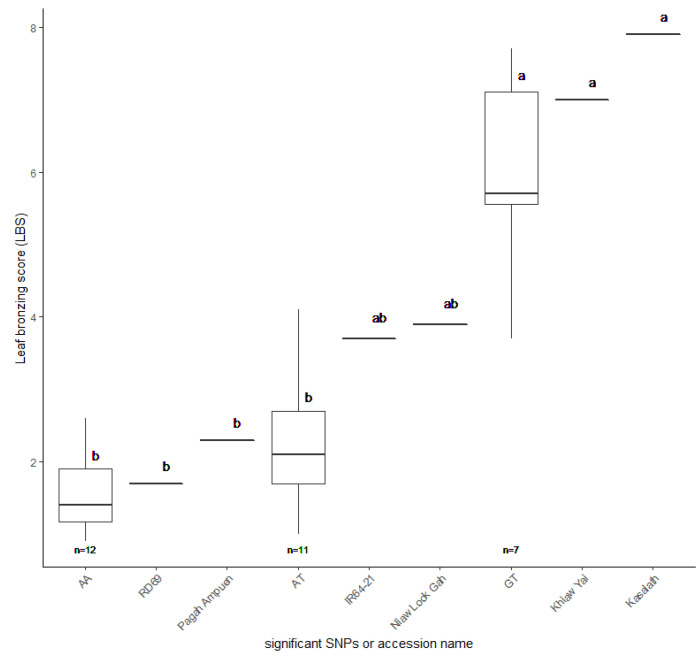
Leaf bronzing score (LBS) of 30 selected Thai rice accessions. RD69 and Pagah Ampuen (AA group) representing tolerance accessions, Khiaw Yai and Kasalath (GT group) representing sensitive accessions, and IR64-21 and Niaw Look Gah (AT group) representing moderate accessions. The LBS values and standard errors were plotted. Letters indicate significant differences between groups at *p* < 0.05 by Tukey’s HSD test.

Additionally, the FT-tolerant accessions with AA combination, RD69 and Pagah Ampuen, and the FT sensitive accessions with GT combination, Khiaw Yai and Kasalath, were included in this experiment. IR64-21 and Niaw look Gah (AT group) represented moderately FT-tolerant accessions. Our results revealed that, based on LBS, AA and AT groups are more FT-tolerant than the GT group (Figure 8; Table S5). It should be noted that the average LBS of AA and AT groups are not significantly different from each other. Together, these results suggest that the LBS associated with SNP:2_21262165 may play a significant role in tolerance to FT in Thai rice.

## 3. Discussion

This study focuses on identifying candidate loci for FT tolerance from seedlings of a highly diverse Thai rice population. Although FT can affect rice growth at any stage, its most harmful effects are found on young seedlings, resulting in complete crop failure [8]. This study applied FT stress to seedlings and evaluated LBS, chlorophyll content, SH, RL, SDW and RDW. Several previous studies demonstrated that LBS symptoms are correlated with FT tolerance [17,23,24,25,37]. Our results showed that LBS demonstrated a strong negative correlation with chlorophyll content (Figure 2). The tolerant accessions still maintained high chlorophyll content and green leaf under FT. The drastic drop of chlorophyll contents found in FT-sensitive accession could result from oxidative stress damage of cellular components that caused photosynthetic reduction [3,52]. This study revealed that rice growth and biomass characters firmly declined under FT stress (Figure 1a–c). However, root dry weight was minimally affected by FT stress (Figure 1d); this could be a result of Fe plaque formation at the root surface, which is a mechanism for excessive Fe exclusion [8,53]. Interestingly, Mathus et al. (2015) and Zhang et al. (2017) also reported an increase in RDW under FT stress, supporting the idea that FT could induce root Fe plaque formation [35,37]. However, our results revealed that the majority of the FT-tolerant accessions (LBS below the median) demonstrated higher SPAD and SDW ratios than the sensitive accessions (Figure S3), implying that the root Fe plaque formation is not a significant mechanism responsible for FT tolerance in this rice population.

Finding the genetic loci regulating the FT-tolerant trait is crucial for developing FT-tolerant rice accessions. Association mapping utilized historical recombination and mutation events within a population to accurately detect marker–trait association [54]. Nevertheless, association mapping can suffer from false-positive results due to population structure and family relatedness [55]. Therefore, the selection of an appropriate mapping population is essential for GWAS. In this study, most Thai rice accessions belong to the indica subpopulation (Figure 3). We decided to obtain indica SNPs through re-alignment to 93-11 indica reference genome and removed the japonica subpopulation (Figure S4). GWAS using indica SNPs identified 16 significant SNPs associated with FT tolerance (Table S3). Comparison of the GWAS results from indica and japonica SNPs data revealed that SNP:2_21262165, the highest significant SNP for LBS trait on chromosome 2, was lost when using japonica SNPs (Figure 6a; Table 2). This emphasizes the influence of the reference genome’s choice on identifying FT-tolerant candidate loci discovery by GWAS. However, the significant SNPs identified by this study have no effects on gene structure and function (Table S4). The use of indica reference genome for SNP calling may provide more accurate gene information for the identification of putative candidate genes (Table 2).

The candidate genes in LD decay (±100 kb) of three significant SNPs (SNP:2_21262165, 1_31789648, and 11_3412238) associated with LBS are related to cellular redox homeostasis (Table 3). Enhanced detoxification and partitioning of Fe^2+^ had already been described as a shoot-base tolerant mechanism [56]. The highest significant SNP associated with LBS, SNP:2_21262165, has two candidate genes closely located in the same LD block. These two genes are involved in carotenoid biosynthesis (*BGIOSGA006309*) and innate immune response (*BGIOSGA006308*). Carotenoids can prevent lipid peroxidation through reactions with lipid peroxyl radicals [57] and protect cellular or subcellular from the ROS effects [58]. However, Turhadi et al. (2018) and Wu et al. (2017) suggested that carotenoid content is unconnected FT because carotenoids only react with singlet oxygen (^1^O_2_) that is not generated by excess Fe [22,59]. Overload of Fe merely affects carotenoid and chlorophyll content reductions [60], which are essential pigments to photosynthesis. Taken together, carotenoid biosynthesis may not be directly associated with FT tolerance. The second candidate gene, *BGIOSGA006308*, encodes for RAR1 protein (REQUIRED FOR MLA12 RESISTANCE 1). Previous studies have reported that RAR1 functions in plant innate immune response to multiple pathogen attacks via interacting with HSP90 (HEAT SHOCK PROTEIN 90) and SGT1 (SUPPRESSOR OF THE G2 ALLELE OF SKP1) [61,62,63,64,65,66,67,68,69]. In rice, *osrar1* loss of function mutant displayed a loss of pathogen immunity [70], while overexpression of *OsRar1* increased basal disease resistance [69]. RAR1 interacts with the N-terminal ATPase domain (ND) of HSP90 to form a chaperone complex for stabilization of resistance (R) genes [62]. Although, the role of the RAR1/HSP complex on abiotic stress response has not been characterized. Several studies have found HSP90 to be involved in abiotic stress responses, including heavy metals toxicity in rice [70,71,72,73,74,75,76,77]. Altogether, these data suggest that the synergy of RAR1 and HSP90 may enhance FT tolerance in rice by improving cellular redox homeostasis. Another candidate gene in the SNP:1_31789648 LD block is the glutathione transferase gene (BGIOSGA000987). Similarly, Matthus et al. (2015) performed GWAS of FT tolerance in rice [37]; they identified this candidate gene and demonstrated that its expression could be strongly induced by FT stress.

In summary, our study identified 16 candidate loci for FT tolerance from highly diverse indica Thai rice accessions. The presence of the candidate SNPs was validated in other FT-tolerant indica Thai rice accessions. SNP:2_21262165 may play a significant role in the shoot-based FT tolerance mechanism in Thai indica rice. Future research may focus on the functional characterization of candidate genes in this region and developing SNP markers for molecular breeding programs.

## 4. Materials and Methods

### 4.1. Rice Population and Fe Toxicity Experiment

The population was composed of 240 Thai rice accessions, landrace and inbred accessions covering all ecosystems, such as upland, rainfed lowland, irrigated, and deepwater rice. Thirty representative accessions, including 22 accessions from the rice diversity panel 1 (RPD1) and eight accessions as international parents from the Thailand Rice Department’s breeding program, were included. The 22 RPD1 accessions were classified into five subpopulation groups, including eight indica, five tropical japonica, three temperate japonica, two aromatic accessions, and three Aus-type accessions, including one admixture accession [78]. The list of accessions can be found in Table S1.

Screening experiments were conducted in a hydroponic system with three replications. Each replication was conducted 1–2 weeks interval in the greenhouse of the Thailand Rice Science Institute (TRSI) from January to March 2019. The average temperature in the greenhouse ranged from 29–40 °C (6.00 a.m.–6.00 p.m.) and 23–29 °C (6.00 p.m.–6.00 a.m.). Seeds of each sample in each replication were germinated on tissue paper in a plastic box for ten days. Twelve uniform plants were transferred to hydroponic solution (1.4 mM KNO_3_, 0.6 mM NaH_2_PO_4_·2H_2_O, 0.5 mM K_2_SO_4_, 0.8 mM MgSO_4_, 0.2 mM CaCl_2_·6H_2_O, 0.07 mM Fe-EDTA, 0.009 mM MnCl_2_·4H_2_O, 0.0001 mM (NH_4_)_6_Mo_7_O_24_·4H_2_O, 0.037 mM H_3_BO_3_, 0.0003 mM CuSO_4_·5H_2_O, 0.00075 mM ZnSO_4_·7H_2_O, pH 5.5), which was modified from Hubbart et al. [79]. For each accession, six plants were subjected to high Fe conditions. Each container contained 12 different accessions, which were randomly arranged. Plants were fixed with sponges into the hole on the lid of the hydroponic container. The solution was renewed twice a week. Twenty-one-day-old seedlings were supplemented with 1000 ppm Fe^2+^ as FeSO_4_ × 7H_2_O for the high Fe treatment. After three days, the phenotypic data, collated as an LBS, was scored using the standard evaluation system for rice (SES), which ranged from 0 (healthy leaf) to 9 (dead or dying leaf) [80]. The SPAD was measured using a chlorophyll meter (SPAD-502Plus, Minolta) three days after the high Fe treatment. Additionally, the SH and RL of each plant were measured five days after stress. After this, the samples were dried at 70 °C for three days and then weighed. SDW and RDW were recorded. All six traits were measured for Fe treated plants and control.

### 4.2. Phenotypic Data Analysis

The effects of treatment, genotype, and the interaction of both were analyzed by two-way analysis of variance (ANOVA) using RStudio v1.2.1335 [81]. The ratio of each trait was calculated by phenotypic data in the high Fe condition/phenotypic data in the control condition. Tukey’s HSD test was applied to the means of each trait. The phenotypic data relationships were determined by Pearson’s correlation (r) using the “corrplot” package [82] and linear regression in RStudio.

### 4.3. Japonica SNP Genotyping and Subpopulation Analysis

The genotypic data as BAM files from Ion S5™ XL Sequencer (Thermo Fisher Scientific) were generated by the Ubon Ratchathani rice research center, Rice Department, Thailand. The DNA library was prepared using the *ApeKI* enzyme for genomic DNA digestion. DNA fragments were ligated with adaptors and then selected for 250–300 bp using E-Gel™ SizeSelect™ agarose gels (Invitrogen) for sequencing. The sequencing data were aligned against the Nipponbare genome as the japonica reference genome by Ion Torrent™ Suite Software Alignment Plugin v5.2.2. The BAM files were converted to fastq files using Samtools v1.9 [83] and realigned with the japonica reference genome using Burrow–wheeler aligner (BWA) v0.7.17 [84] and SAMtools, respectively. Variants were called using a genome analysis toolkit (GATK) v4.1.4.1 [85] and removed the SNPs that showed heterozygous allele, minor allele frequency (MAF) of less than 0.05, and missing data of more than 0.5 using VCFtools v0.1.13 [86]. These filtered SNPs were called “japonica SNPs”.

The subpopulation was confirmed by comparison with rice accessions in the 3000 rice genomes project (3KRGP). The japonica SNPs that had missing data of less than 0.05 were selected to generate “core SNPs”. Subsequently, the core SNPs of 3KRGP were selected and then removed from the missing data accessions. The core SNPs of the two populations were merged using VCFtools and continued analysis by principal component analysis (PCA) using PLINK v1.9 [87].

### 4.4. Indica SNP Genotyping and Population Structure Analysis

The fastq files of 270 accessions were aligned against the 93-11 indica reference genome using BWA. SAM files were obtained using SAMtools. SNPs were called based on previous criteria for GWAS. Population structure was analyzed by PCA using PLINK. Population stratification was visualized by plotting the first two PCs using RStudio. The population was divided into two groups as group 1 (G1: genetic group close to indica type) and group 2 (G2: genetic group close to japonica type) by comparing with RPD1 accessions. To reduce population structure, accessions that fell out of the main group were removed to generate accurately significant SNPs by VCFtools.

### 4.5. Association Mapping and Linkage Disequilibrium (LD) Analysis

Genome-wide association (GWA) mapping was performed in the indica subpopulation (G1) using factored spectrally transformed linear mixed models (FaST-LMM-v1.08) [88]. Indica SNPs and LBS, the ratio of SPAD, SH, RL SDW and RDW were conducted to identify significant SNPs associated with phenotypic data. The *p*-value of the SNP marker was corrected for multiple tests by the *q*-value (FDR adjusted *p*-value) of each trait. SNPs with a *q*-value lower than 0.05 were selected as significant markers. The association mapping results were presented in Manhattan plots constructed from SNPs positions and −log10 (*p*-value) of each SNP and quantile–quantile (Q–Q), which were generated from observed and expected *p*-values using R “CMplot” package [89] in RStudio. To compare indica and japonica SNPs, the japonica SNP’s association mapping was also conducted with the six traits.

LD in indica subpopulation was calculated using the correlation (*r*^2^) between a pair of SNPs loci within 2000 kb using PopLDdecay [47]. The *r*^2^ value within 20 kb was averaged to estimate LD decay and was plotted against the physical distance using the ‘ggplot2′ package [90] in RStudio. The distance at average *r*^2^ dropped to half of the maximum value was described as LD decay. Candidate genes were considered from the list of genes in the LD decay of significant SNPs.

### 4.6. SNP Validation in Other Thai Rice Accessions

For validation, 30 Thai rice accessions were selected from another population. These accessions were evaluated under control and high Fe conditions at the seedling stage. The average day and night temperatures in May (2020) under greenhouse conditions of TRSI were from 32 to 40 and 28 to 35 °C. The average phenotype was compared between candidate SNP groups using Tukey’s HSD test and plotted using the ‘ggplot2′ package in RStudio.

## Figures and Tables

**Table 1:** Descriptive statistics and ANOVA results for six phenotypes derived from 270 accessions.

Trait	Control			Treatment (1000 ppm Fe^2+)^			ANOVA Result		
	Min	Max	Mean ± SD	Min	Max	Mean ± SD	G	T	G*T
Leaf bronzing score	0	0	0	0.7	9.0	2.7 ± 1.72	NA	NA	NA
Chlorophyll content (SPAD value)	13.40	30.88	22.95 ± 3.11	10.06	32.24	22.26 ± 3.28	***	***	***
Shoot height (cm)	10.06	32.24	45.55 ± 6.93	22.57	62.51	33.68 ± 5.20	***	***	***
Root length (cm)	16.33	39.87	23.62 ± 3.87	12.52	29.17	19.23 ± 2.87	***	***	***
Shoot dry weight (g)	0.0441	0.2386	0.1256 ± 0.0370	0.0371	0.1703	0.0830 ± 0.0217	***	***	***
Root dry weight (g)	0.0116	0.0685	0.0339 ± 0.0101	0.0168	0.0786	0.0344 ± 0.0096	***	**	***

*p* < 0.001 (***) and *p* < 0.01 (**). NA, not applicable; Min, minimum; Max, maximum; SD, standard deviation; G, genotype; T, treatment; G*T, genotype and treatment interaction.

**Table 2:** The comparison of significant SNPs found by association analysis using indica and japonica SNPs.

No.	Trait	Indica SNP						Japonica SNP					
		SNP	Chr.	Position (bp)	*q*-value	REF	ALT	SNP	Chr.	Position (bp)	*q*-value	REF	ALT
1	LBS	2_21262165	2	21,262,165	0.02	A	G						
		1_31789648	1	31,789,648	0.02	T	A	1_28485029	1	28,485,029	0.03	A	T
		11_3412238	11	3,412,238	0.03	C	T						
2	SPAD ratio	5_11219514	5	11,219,514	0.01	T	A	5_9383108	5	9,383,108	0.01	A	T
		5_11219586	5	11,219,586	0.01	G	A						
3	SDW ratio	1_30038228	1	30,038,228	0.01	T	C	1_26826635	1	26,826,635	0.01	C	T

**Table 3:** Putative candidate genes located in ± 100 Kb region of significant SNPs.

SNP	Indica Gene ID	MSU Gene ID	Start Position (bp)	Stop Position (bp)	Description of Function *
2_21262165	BGIOSGA006309	LOC_Os02g33149	21,278,308	21,281,517	positive regulation of the carotenoid biosynthetic process
	BGIOSGA006308	LOC_Os02g33180	21,290,620	21,292,002	defense response to bacterium, plant-type hypersensitive response, respiratory burst involved in defense response
1_31789648	BGIOSGA000987	LOC_Os01g49720	31,878,687	31,879,477	glutathione metabolic process
	BGIOSGA004247	LOC_Os01g49740	31,882,740	31,884,784	chloroplast accumulation movement, chloroplast avoidance movement
11_3412238	BGIOSGA034416	LOC_Os11g07280	3,446,793	3,451,671	intracellular protein transport/vesicle-mediated transport
5_11219514 5_11219586	BGIOSGA019494	LOC_Os05g16630	11,265,443	11,268,553	Thioredoxin domain-containing protein
1_30038228	BGIOSGA004143	LOC_Os01g46940	29,991,866	29,993,257	lipid biosynthetic process

* Gene description or function was obtained from UniProt Knowledgebase (UniProtKB) [51].

## Data Availability

The data presented in this study are available in Supplementary Materials.

## Supplementary Materials

The following are available online at https://www.mdpi.com/article/10.3390/plants10040798/s1, Figure S1: Leaf bronzing score (LBS) was assessed from 0 (healthy plant) to 9 (dead or dying plant) at day three after applying 1000 ppm Fe^2+^. The high tolerance accessions (score 0–1) showed normal growth, whereas sensitive accessions (score 5–9) showed reddish-brown spots and discolored leaves or dying; Figure S2: Frequency distribution of (a) leaf bronzing score (LBS), (b) the ratio of SPAD value, (c) the ratio of shoot height, (d) the ratio of root length, (e) the ratio of shoot dry weight, and (f) the ratio of root dry weight. For LBS, Taichung Native 1 and Kasalath, representing accession from rice panel diversity 1 (RPD1), showed high tolerance and sensitivity for iron toxicity (FT), respectively. According to a previous study, Azucena, Nipponbare, and Pokkali were moderate varieties, whereas IR64 was a sensitive variety; Figure S3: The relationships between leaf bronzing score (LBS) and the ratio of SPAD value and shoot dry weight were analyzed using linear regression. Horizontal and vertical dashed red lines represent the median values of each trait. The linear equation and R^2^ in each relationship were shown in the graph. (a) Linear regression of LBS versus the ratio of SPAD value (b) Linear regression of LBS versus the ratio of shoot dry weight; Figure S4: The population structure of 270 accessions was analyzed using 73,054 indica SNPs by PCA, divided into two main groups: group 1 (blue dot; indica) and group 2 (plink dot; japonica); Figure S5: The number of (a) indica and (b) japonica SNPs within 1 Mb window size in 12 chromosomes of 229 accessions; Figure S6: Manhattan and quantile–quantile (Q–Q) plot of GWAS using 41,178 indica SNPs associated with a) the ratio of shoot height, b) the ratio of root length (RL), and c) ratio of root dry weight (RDW) from 229 accessions (group 1) using FaST-LMM. There were no significant SNPs identified by *q* value < 0.05 threshold; Figure S7: Comparison between chr1:26,726,635–26,926,635 (japonica) and chr1:29,938,228–30,138,228 (indica) syntenic regions; Figure S8: Comparison between chr1:28,385,029–28,585,029 (japonica) and chr1:31,689,648–31,889,648 (indica) syntenic regions; Figure S9: Comparison between chr5:9,283,108–9,483,108 (japonica) and chr5:11,119,514–11,319,514 (indica) syntenic regions; Figure S10: The population structure of the validation set (30 accessions) and the 270 accessions by principal component analysis; Table S1: Information of 270 accessions used in this study, including subpopulation groups that were identified using 73,054 indica SNPs by principal component analysis; Table S2: The average of trait values observed in both control and stress conditions and the calculated ratio value of 270 accessions; Table S3: The comparison of significant SNPs from association analysis using indica and japonica SNPs in 229 accessions; Table S4: List of genes located in LD block of each significant SNPs and their information, including orthologous in *Oryza sativa* (indica and japonica type) and *Arabidopsis thaliana*; Table S5: List of 30 Thai rice accessions used for SNP validation.

## Abbreviations

FT	Iron (Fe) toxicity
LBS	Leaf-bronzing score
SPAD	Chlorophyll content of expanded leaf that was measured by chlorophyll meter (SPAD value)
SH	Shoot height (cm)
RL	Root length (cm)
SDW	Shoot dry weight
RDW	Root dry weight
RPD1	Rice diversity panel 1
3KRGP	3000 rice genomes project
PCA	Principal component analysis
QTL	Quantitative trait loci
GWAS	Genome-wide association study
FaST-LMM	Factored spectrally transformed linear mixed models
SNP	Single-nucleotide polymorphism
MAF	Minor allele frequency
LD	Linkage disequilibrium
REF	Reference allele
ALT	Alternative allele
Chr	Chromosome
RAR1	REQUIRED FOR MLA12 RESISTANCE 1
HSP90	HEAT SHOCK PROTEIN 90
