# Supplementary figures and images for: Genome-Wide Association Study of Local Thai Indica Rice Seedlings Exposed to Excessive Iron
Source: Plants (Basel). 2021 Apr 19;10(4):798. doi: 10.3390/plants10040798 (PMC8073664; doi:10.3390/plants10040798)

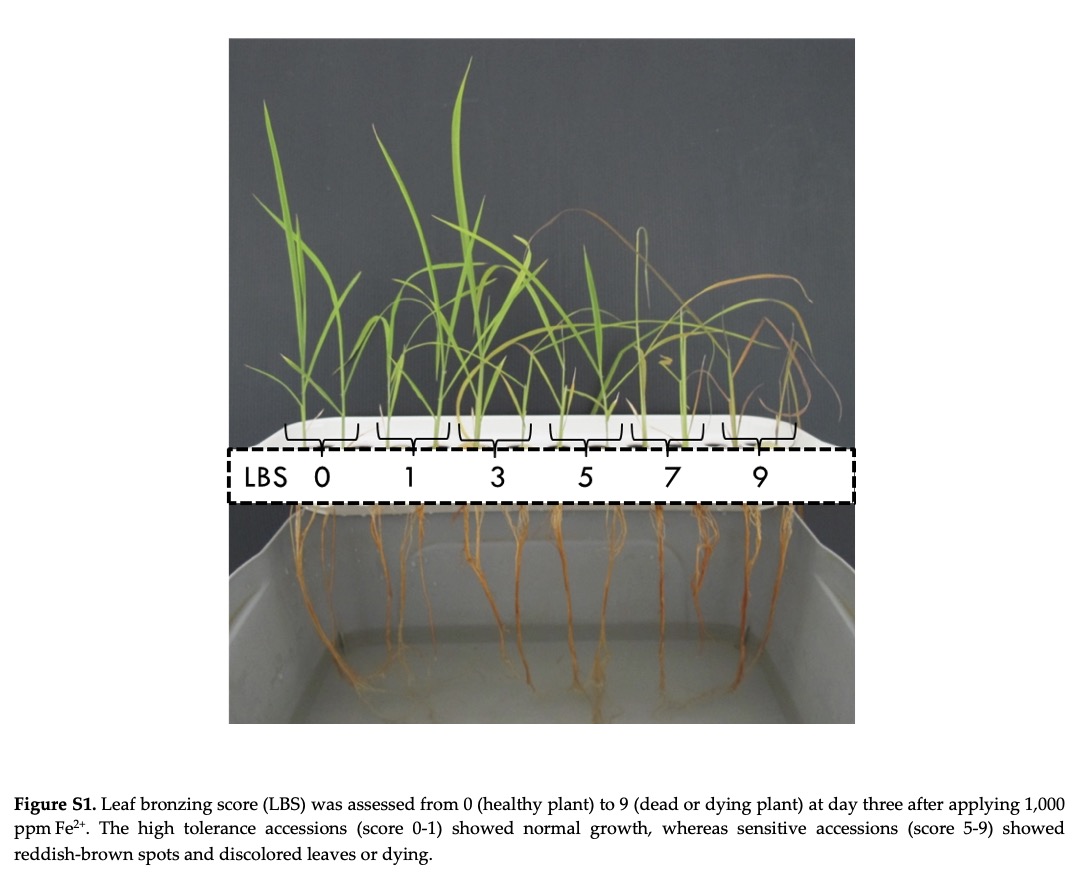

Supplement: Supplementary file 1 [file plants-10-00798-s001.zip › Figure_S1.jpg]

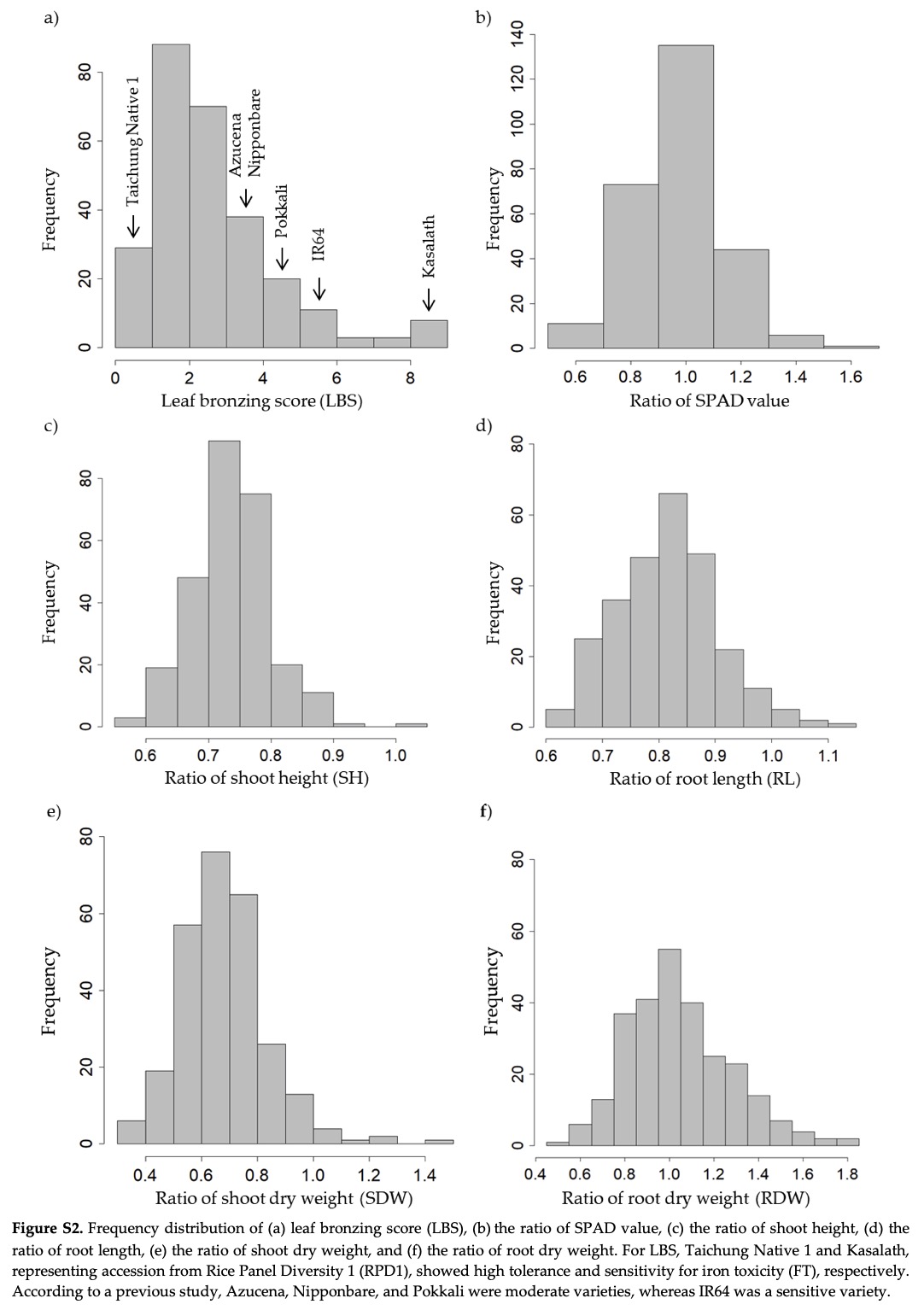

Supplement: Supplementary file 1 [file plants-10-00798-s001.zip › Figure_S2.jpg]

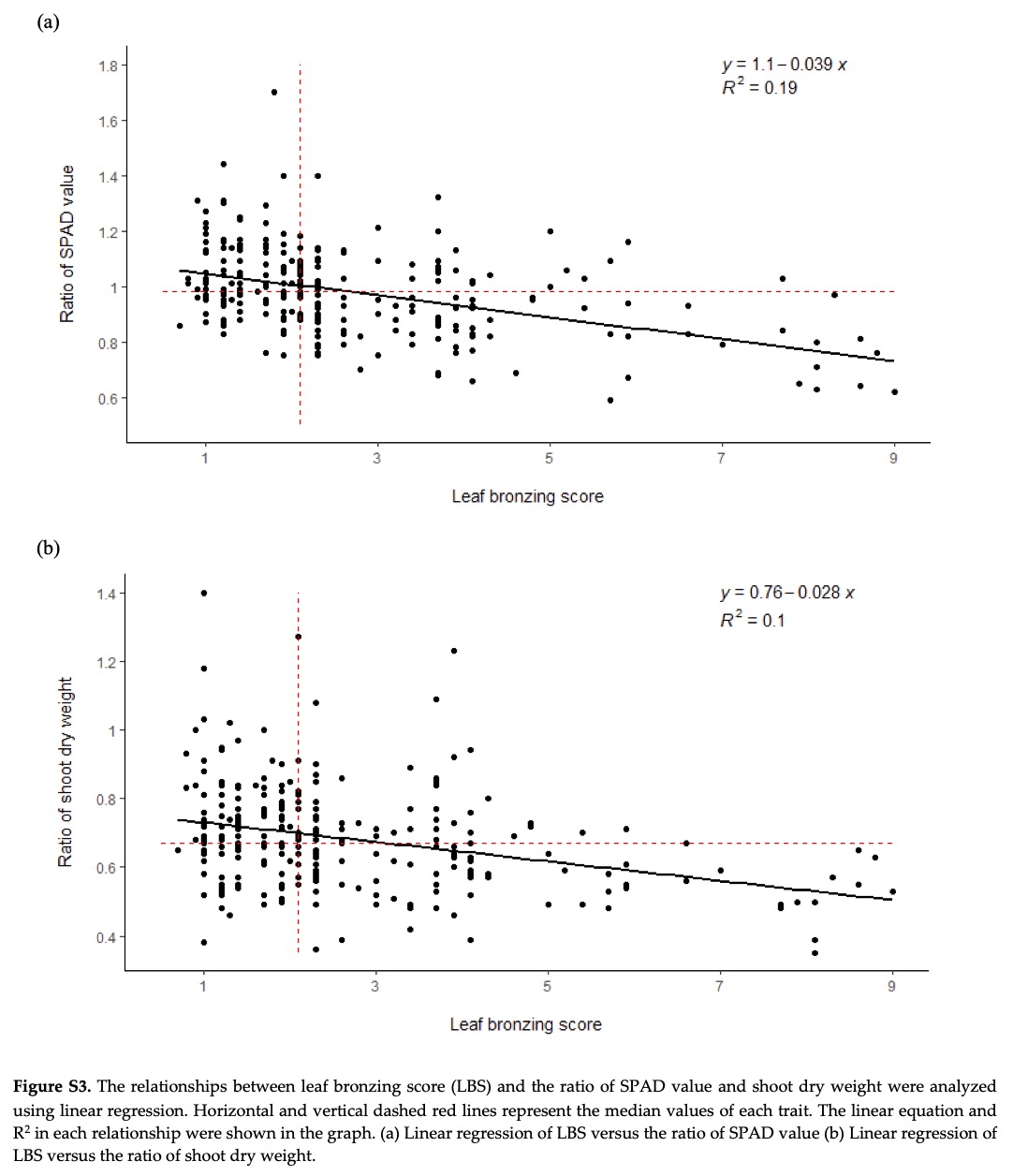

Supplement: Supplementary file 1 [file plants-10-00798-s001.zip › Figure_S3.jpg]

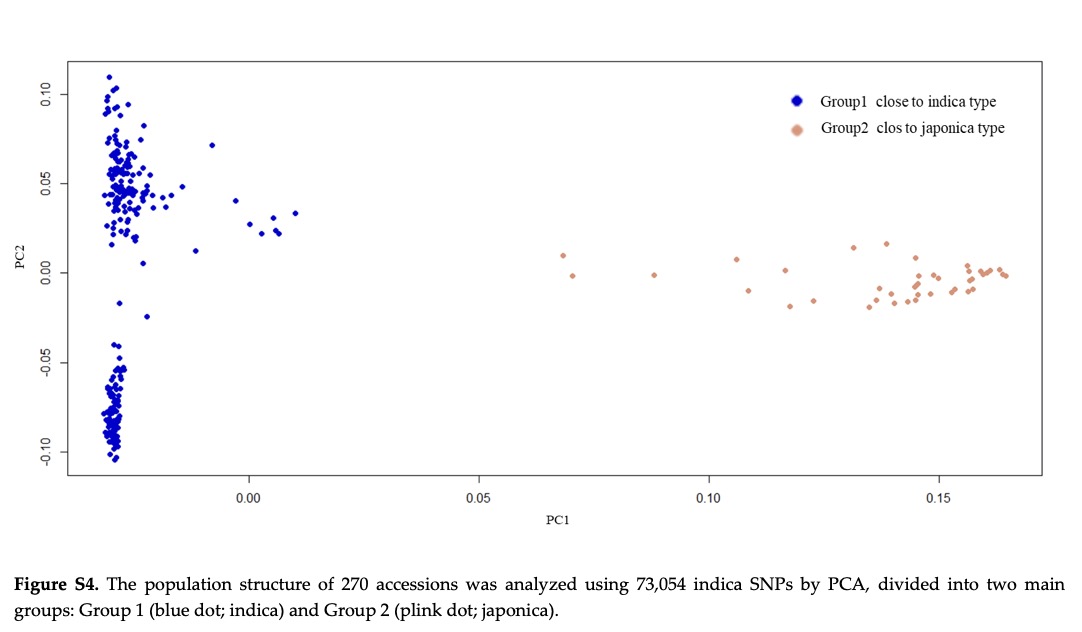

Supplement: Supplementary file 1 [file plants-10-00798-s001.zip › Figure_S4.jpg]

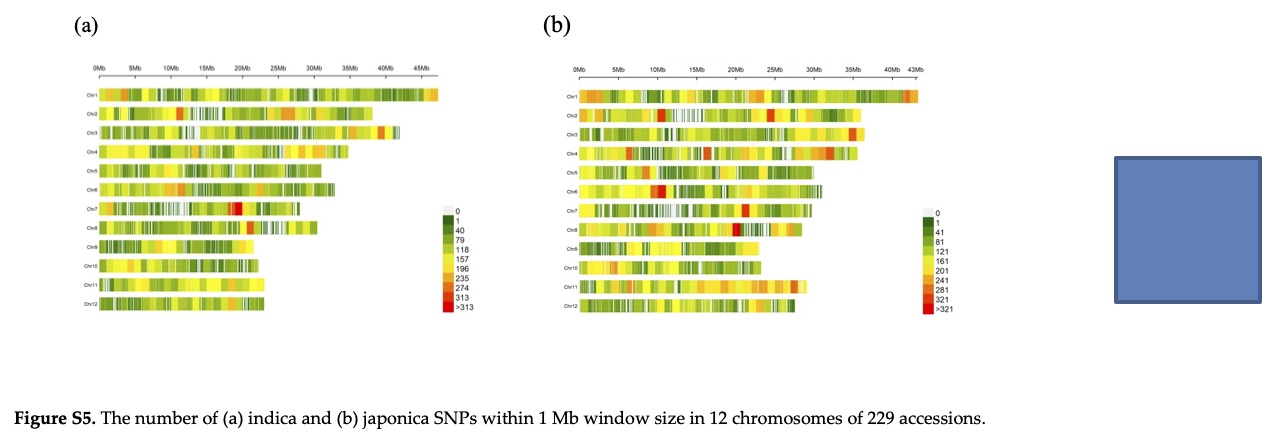

Supplement: Supplementary file 1 [file plants-10-00798-s001.zip › Figure_S5.jpg]

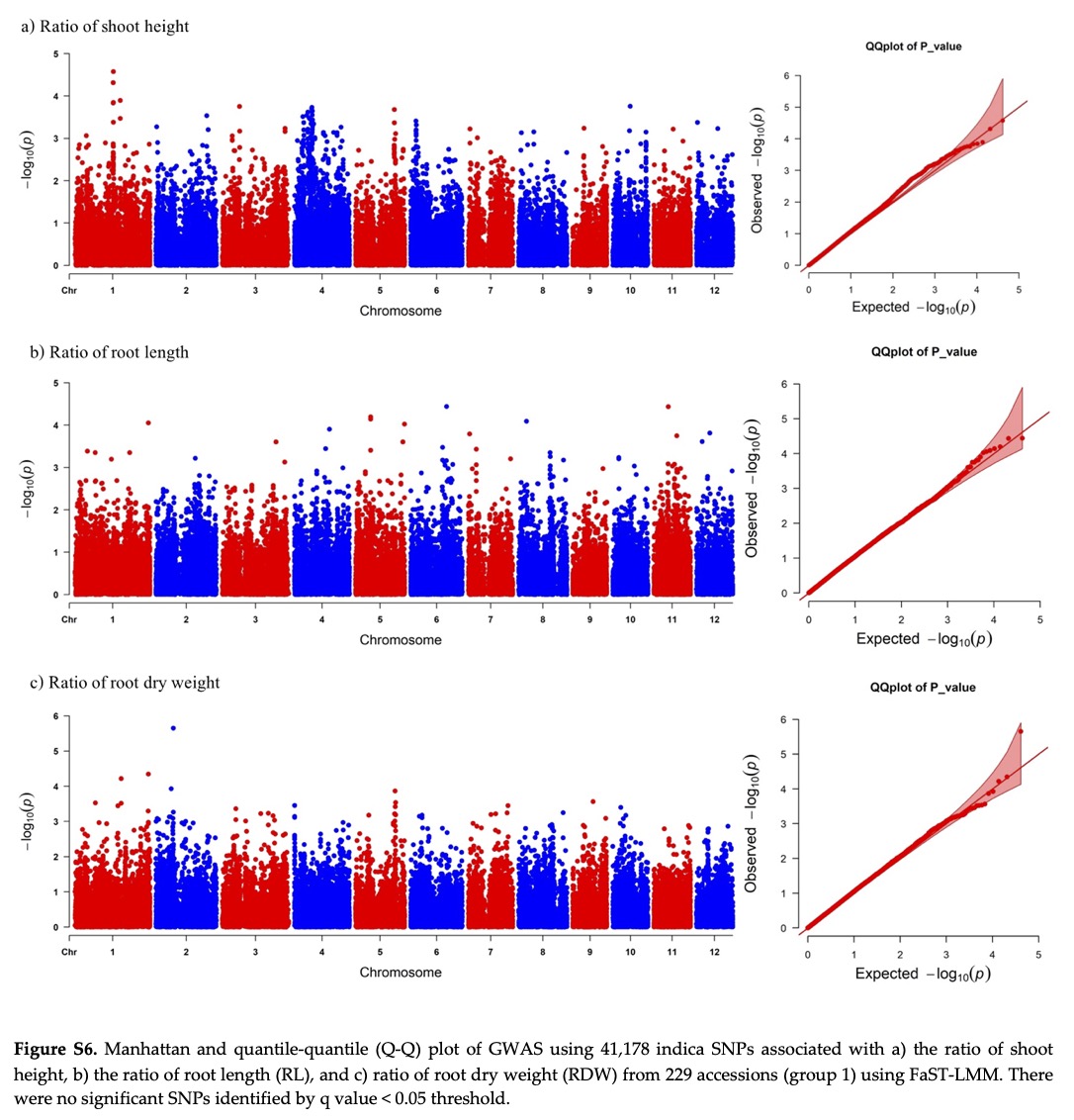

Supplement: Supplementary file 1 [file plants-10-00798-s001.zip › Figure_S6.jpg]

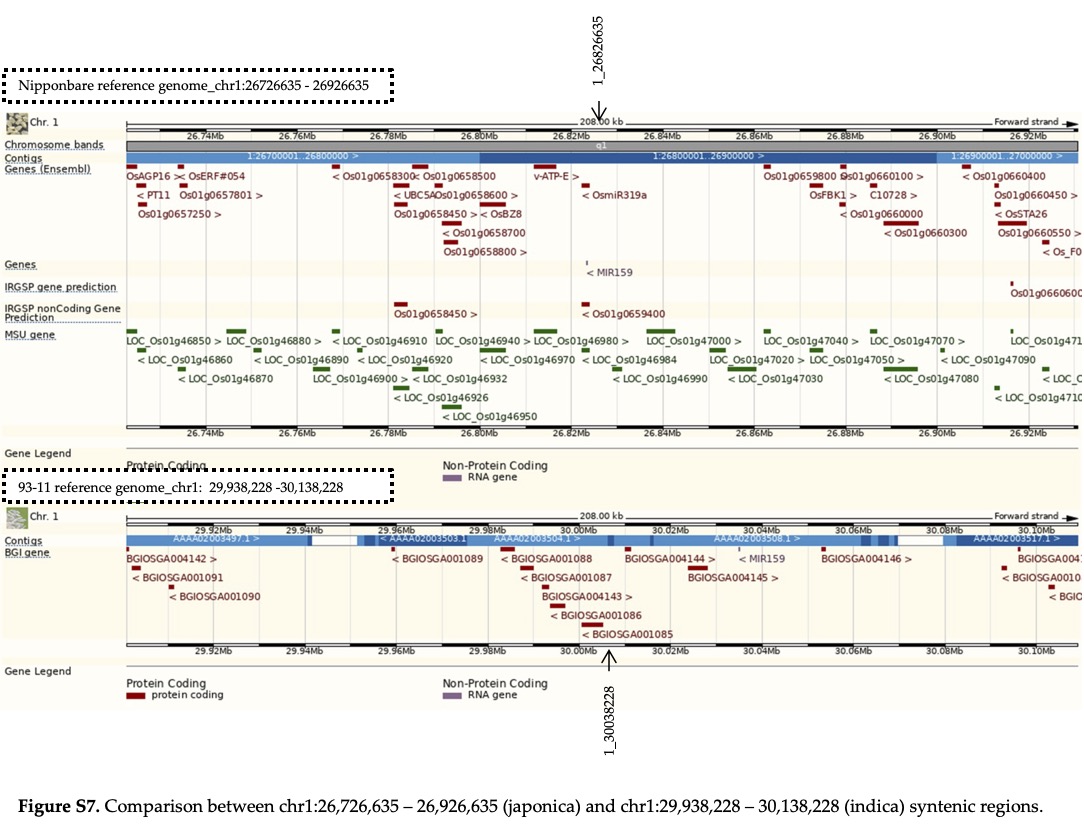

Supplement: Supplementary file 1 [file plants-10-00798-s001.zip › Figure_S7.jpg]

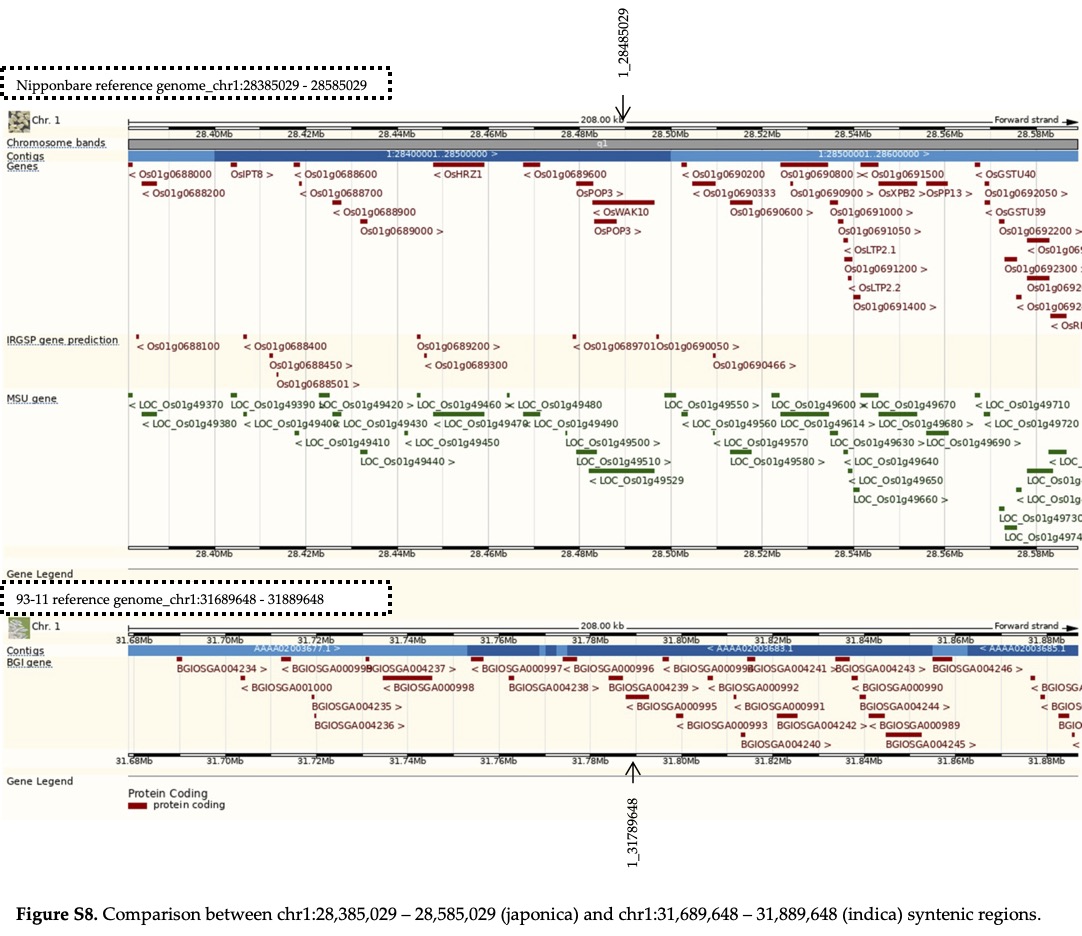

Supplement: Supplementary file 1 [file plants-10-00798-s001.zip › Figure_S8.jpg]

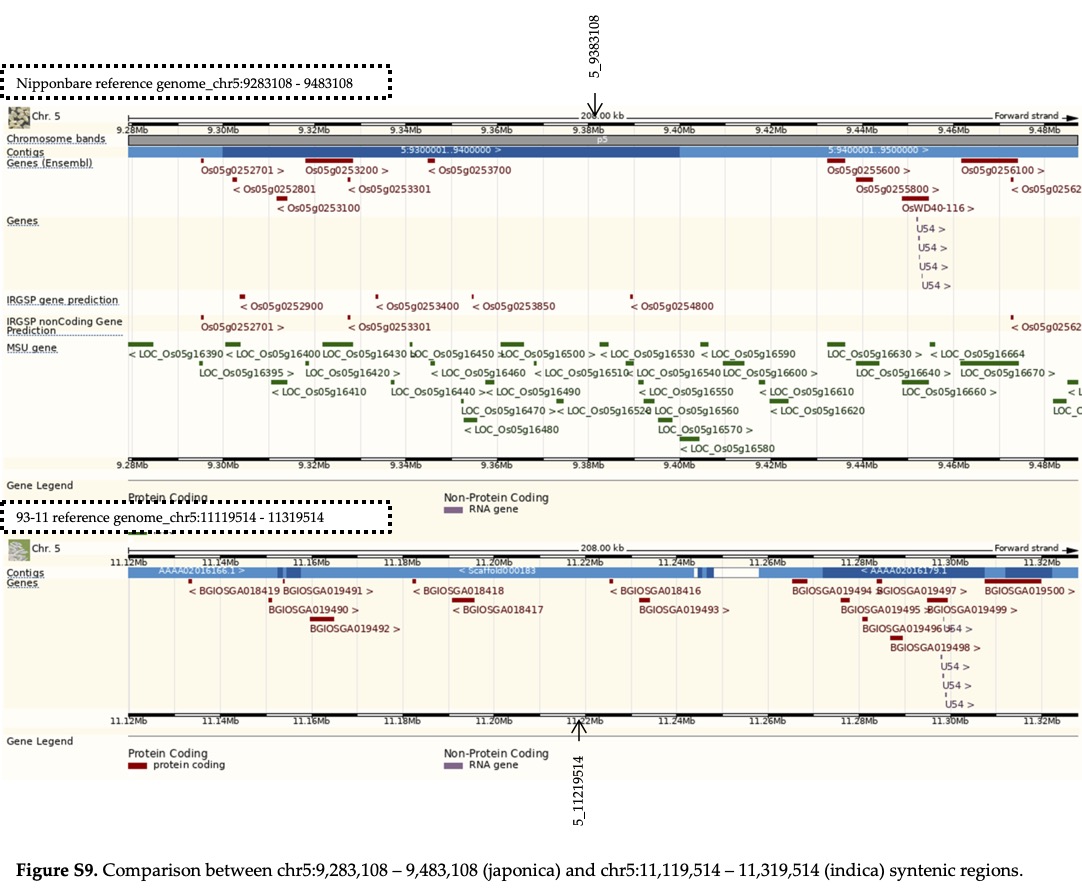

Supplement: Supplementary file 1 [file plants-10-00798-s001.zip › Figure_S9.jpg]

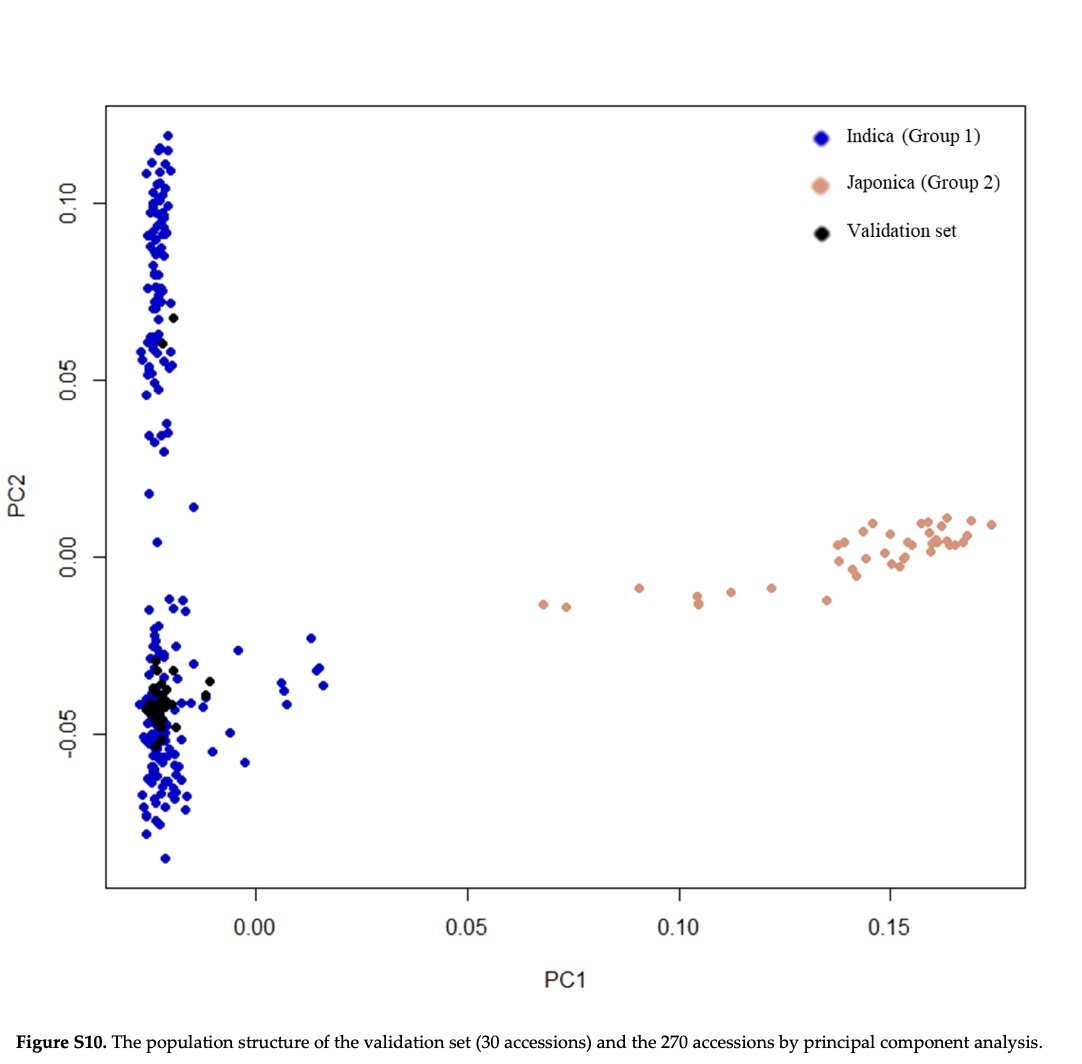

Supplement: Supplementary file 1 [file plants-10-00798-s001.zip › Figure_S10.jpg]
